# *Petrocephalus boboto* and *Petrocephalus arnegardi*, two new species of African electric fish (Osteoglossomorpha, Mormyridae) from the Congo River basin

**DOI:** 10.3897/zookeys.400.6743

**Published:** 2014-04-10

**Authors:** Sébastien Lavoué, John P. Sullivan

**Affiliations:** 1 Institute of Oceanography, National Taiwan University, Roosevelt Road, Taipei 10617, Taiwan; 2 Cornell University Museum of Vertebrates, 159 Sapsucker Woods Road, Ithaca, New York 14850 USA

**Keywords:** Biodiversity, weakly electric fish, Petrocephalinae, integrative taxonomy, phylogeny, cytochrome *b*, electric organ discharge

## Abstract

A specimen of the African weakly electric fish genus *Petrocephalus* (Osteoglossomorpha, Mormyridae) collected in the Congo River at Yangambi, Orientale Province, Democratic Republic of Congo, is described as a new species. *Petrocephalus boboto* **sp. n.** can be distinguished from other Central African species of *Petrocephalus* by a combination of the following characteristics: three distinct black spots on the body, one at the origin of the pectoral fin, one at the origin of the caudal fin and one below the anterior base of the dorsal fin; Nakenrosette and Khelrosette electroreceptor clusters distinct on head but Augenrosette cluster reduced in size; 23 branched dorsal rays, 34 branched anal rays, and electric organ discharge waveform triphasic. *Petrocephalus boboto* **sp. n.** most closely resembles the holotype of *Petrocephalus binotatus* but is easily distinguished from it by its smaller mouth. A comparative molecular analysis including 21 other *Petrocephalus* species shows *Petrocephalus boboto* **sp. n.** to be genetically distinctive and to represent a deep lineage in the genus. Two specimens of *Petrocephalus* collected at Yangambi are morphologically similar and genetically closely related to specimens previously assigned to *Petrocephalus binotatus*, collected in the northwestern Congo River basin within Odzala-Kokua National Park, Republic of the Congo. This prompts us to formally describe a new species from these collections, *Petrocephalus arnegardi* **sp. n.**, that, although similar to the holotype of *Petrocephalus binotatus*, can be distinguished from it by its smaller mouth and shorter interorbital width.

## Abstract (fr)

Un spécimen de poissons africains à faible décharge électrique du genre *Petrocephalus* (Osteoglossomorpha, Mormyridae) collecté dans le fleuve Congo à Yangambi, Province Orientale, République Démocratique du Congo, est décrit comme une nouvelle espèce. *Petrocephalus boboto* **sp. n.** peut-être distinguée des autres espèces de *Petrocephalus* d’Afrique Centrale par la présence de trois distinctes taches noires, l’une à la base des nageoires pectorales, la seconde à la base de la nageoire caudale et la troisième sur chacun des flancs, juste en dessous des premiers rayons de la nageoire dorsale; présence bien visible de deux rosettes électroréceptives sur la tête (Nakenrosette et Khelrosette), la troisième rosette (Augenrosette) est de taille réduite; 23 rayons branchus à la nageoire dorsale et 34 rayons à la nageoire anale; et décharge électrique triphasique. *Petrocephalus boboto* **sp. n.** ressemble le plus à l’holotype de *Petrocephalus binotatus* mais s’en distingue par une bouche nettement plus petite. Une analyse génétique confirme la distinction de *Petrocephalus boboto* sp. n. parmi un échantillon de 21 espèces. Cette analyse montre également que cette nouvelle espèce représente une lignée distincte de *Petrocephalus*. Deux autres spécimens de *Petrocephalus* collectés à Yangambi sont morphologiquement et génétiquement proches de spécimens auparavant identifiés comme appartenant à *Petrocephalus binotatus*, collectés dans le Parc National d’Odzala-Kokua, bassin du Congo, République du Congo. Ceci nous amène à les décrire comme une nouvelle espèce, *Petrocephalus arnegardi* **sp. n.** qui peut-être distinguée de l’holotype de *Petrocephalus binotatus* par une bouche plus petite et un espace inter-orbitaire plus court.

## Introduction

The monophyletic African weakly electric fishes superfamily Mormyroidea (Teleostei, Osteoglossomorpha) contains two families, the Gymnarchidae and Mormyridae, and 212 species (Eschmeyer and Fong 2014). All mormyroids generate and sense weak electric discharges for the purpose of intraspecific electrocommunication and spatial electrolocation using complex electrogenic and electroreceptive organ systems (Moller 1995, Turner et al. 1999, Bullock et al. 2005). While mormyroids are immediately identifiable by their distinctive appearance, considerable morphological, electrophysiological and behavioral diversity has evolved within the group (Arnegard et al. 2010b, Carlson et al. 2011, Rabosky et al. 2013). These differences lead to the recognition of three main lineages: the monotypic Gymnarchidae and the two reciprocally monophyletic mormyrid subfamilies Mormyrinae and Petrocephalinae with 168 and 43 valid species, respectively (Taverne 1972, Sullivan et al. 2000, Eschmeyer and Fong 2014).

Recent comparative studies have revealed significant electrophysiological differences between Mormyrinae and Petrocephalinae (Lavoué et al. 2008, Carlson and Arnegard 2011, Carlson et al. 2011). In particular, Carlson et al. (2011) suggested that differences among lineages in central and peripheral electrosensory anatomies are correlated with higher electric organ discharge (EOD) waveform diversity and greater species richness in Mormyrinae relative to the Petrocephalinae. While Mormyrinae are clearly more speciose than Petrocephalinae, true species diversity is clearly underestimated in both groups. Despite often subtle morphological differentiation among species of *Petrocephalus*, the sole petrocephalin genus, the pace of new species discovery and description has been rapid in recent years, with about one third of *Petrocephalus* diversity having been described in the 21th century (Lavoué et al. 2004, 2010, Lavoué 2011, 2012, Kramer et al. 2012).

*Petrocephalus* currently includes 43 valid species (Eschmeyer and Fong 2014), not including *Petrocephalus balteatus* (for justification, see Daget 2000), which are widely distributed in tropical and subtropical African freshwaters. They are small fishes predominantly found in riverine systems where they are mostly active at dusk. Several morphological synapomorphies support the monophyly of *Petrocephalus* (Taverne 1969, Taverne 1972), as do molecular data (Sullivan et al. 2000).

**Figure 1.**
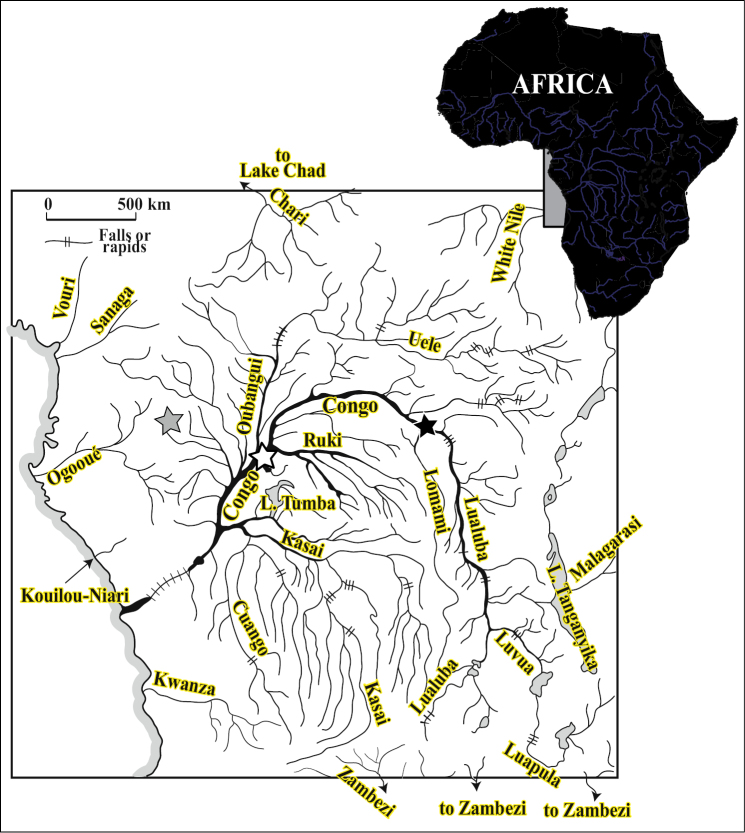
Hydrographic system of the Congo River basin and the type localities of *Petrocephalus boboto* sp. n. (black-filled star) at Yangambi, Democratic Republic of Congo, *Petrocephalus arnegardi* sp. n. (gray-filled star) at Odzala-Kokua National Park, Republic of the Congo and *Petrocephalus binotatus* (white-filled star) at Ikengo, near Mbandaka, Democratic Republic of Congo.

Central African *Petrocephalus* are particularly diverse and abundant throughout the large Congo basin with 18 species (Lavoué et al. 2010, Lavoué 2012). Here, we first describe a new species of *Petrocephalus* from the main channel of the Congo River at Yangambi, Central Congo basin. Based on our long-term work on *Petrocephalus*, we believe this new species is rare, as we have identified only one specimen after examination of hundreds of Central African *Petrocephalus* specimens. From the same locality, we examined two specimens of *Petrocephalus* that share more similarities to the specimens earlier identified as *Petrocephalus binotatus* from Odzala-Kokua (Lavoué et al. 2010) than to the holotype of *Petrocephalus binotatus*, despite the greater proximity of Yangambi to the type locality of *Petrocephalus binotatus* (Ikengo), than to Odzala-Kokua (Fig. 1). This led us to reevaluate this identification and describe the Odzala-Kokua and Yangambi specimens as new.

## Materials and methods

*Petrocephalus* specimens newly examined in this study were collected in September 2010 from the Congo River at Yangambi, Orientale Province, Democratic Republic of Congo (Fig. 1). Comparative material including type specimens from all valid species from the Congo River basin and the Lower Guinea province is listed in Lavoué et al. (2004, 2010) and Lavoué (2012). About half of the nucleotidic sequences of the cytochrome *b* gene (about 600 base pairs from the 3’ end) were determined for three specimens of *Petrocephalus* collected at Yangambi.

In the field, we recorded EODs from two of these three specimens of *Petrocephalus*. Each individual was first transferred to a plastic tank (ca. 80 cm long*30 cm wide*30 cm deep) filled with water from the capture location. EODs were recorded with silver/silver-chloride electrodes connected to an Edirol FA-66 analog-to-digital Firewire audio interface (Roland Corporation). Signals were visualized and stored in SignalScope 2.0 virtual oscilloscope software (Faber Acoustical) such that positivity at the fish’s head was always recorded in the upward direction. Water temperature of each recording was noted. Fish were euthanized with an overdose of MS222, photographed, tissued, tagged with a unique specimen number, fixed in buffered 10% formalin and later transferred to 70% ethanol.

Total peak-to-peak amplitudes of all EODs were normalized to a value of one. We did not correct the time base of EODs to a uniform temperature as this procedure produces only minimal changes. Seven EOD measures were taken as described in Lavoué et al. (2008): relative height of peak 1, relative height of peak 2, relative height of peak 3 (when present), duration of peak 1, duration of peak 2, duration of peak 3 (when present), total EOD duration. EOD recordings have been archived in the Macaulay Library at the Cornell Lab of Ornithology (CUML accession numbers provided in Suppl. material 1).

Mature male specimens were identified by the presence of an anal fin notch (Pezzanite and Moller 1998). Methods for making counts and measurements and their abbreviations follow those given by Boden et al. (1997), except for body height and standard length that are modified as in Lavoué et al. (2004). Furthermore, the snout length and the mouth position are the nearest distances, parallel to the body axis, from the perpendicular line through the tip of the snout to the anterior border of the eye and the corner of the mouth, respectively.

We examined the specimens for the presence of each of the three distinct clusters of Knollenorgan-type electroreceptors on the head of *Petrocephalus*, as described in Harder (1968, 2000). The “Augenrosettes” are directly above the anterior half of the eye, the “Nackenrosettes” are dorsally situated on each side of the nape, slightly anterior to the opercular openings and the “Kehlrosettes” are situated anterior to and extending somewhat below the insertion of the pectoral fins.

DNA was extracted from fin clips preserved in 90% ethanol. PCR amplification and sequencing of the partial cytochrome *b* gene were as described by Sullivan et al. (2000) using the following *Petrocephalus*-specific primers: L15213_MOR (5’-CTA ACC CGA TTC TTT GCC TTC CAC TTC CT-3’) and H15913_MOR (5’-TCG ATC TCC GGA TTA CAA GAC CG-3’). Cytochrome *b* sequences generated in this study are available in GenBank under accession numbers KF181719 to KF181721. The three new partial cytochrome *b* sequences were added to the dataset published in Lavoué (2012) from which individuals uninformative for interspecific comparisons have been removed. Following the conclusions of Kramer et al. (2012) who resurrected several species previously synonymized with *Petrocephalus catostoma*, we assigned revised names to two specimens (and their corresponding cytochrome *b* sequences) treated in Lavoué (2012): one specimen of *Petrocephalus catostoma* from the Rufiji River is renamed *Petrocephalus steindachneri* and one specimen of *Petrocephalus catostoma* from the Wami River is renamed *Petrocephalus stuhlmanni*. The alignment does not require any indels and missing data were coded with “-”. The final alignment comprises 1140 nucleotidic positions. The maximum likelihood phylogenetic tree was calculated under the general time reversible model of nucleotide substitution with rate heterogeneity following a discrete gamma distribution (GTR+Г), using the software RAxML-VI-HPC (Stamatakis 2006) and its graphical interface raxmlGUI (Silvestro and Michalak 2012).

## Taxonomy

### Petrocephalus boboto sp. n.

http://zoobank.org/A0EE9192-B7D4-4565-A598-E251D86AD5D4

http://species-id.net/wiki/Petrocephalus_boboto

**Figure 2.**
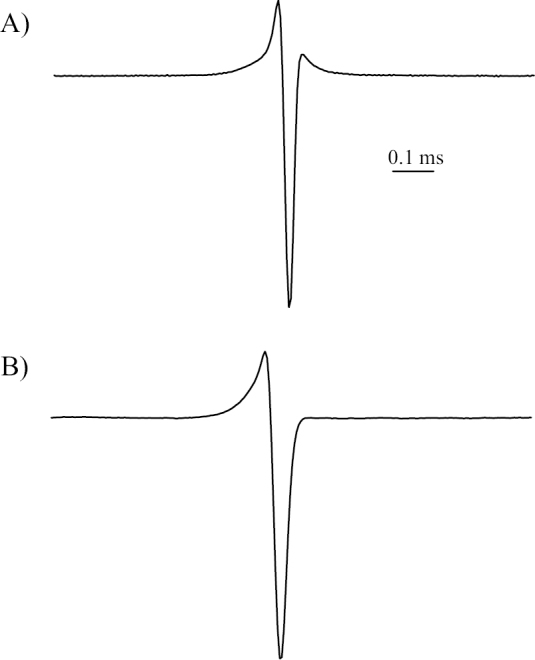
**A** EOD waveform of the holotype of *Petrocephalus boboto* sp. n. from Yangambi (CUMV 96774) **B** EOD waveform of the specimen JPS-511 of *Petrocephalus arnegardi* from Yangambi (BMNH 2013.8.29.125). Waveform plotted with head-positivity upwards.

**Figure 3.**
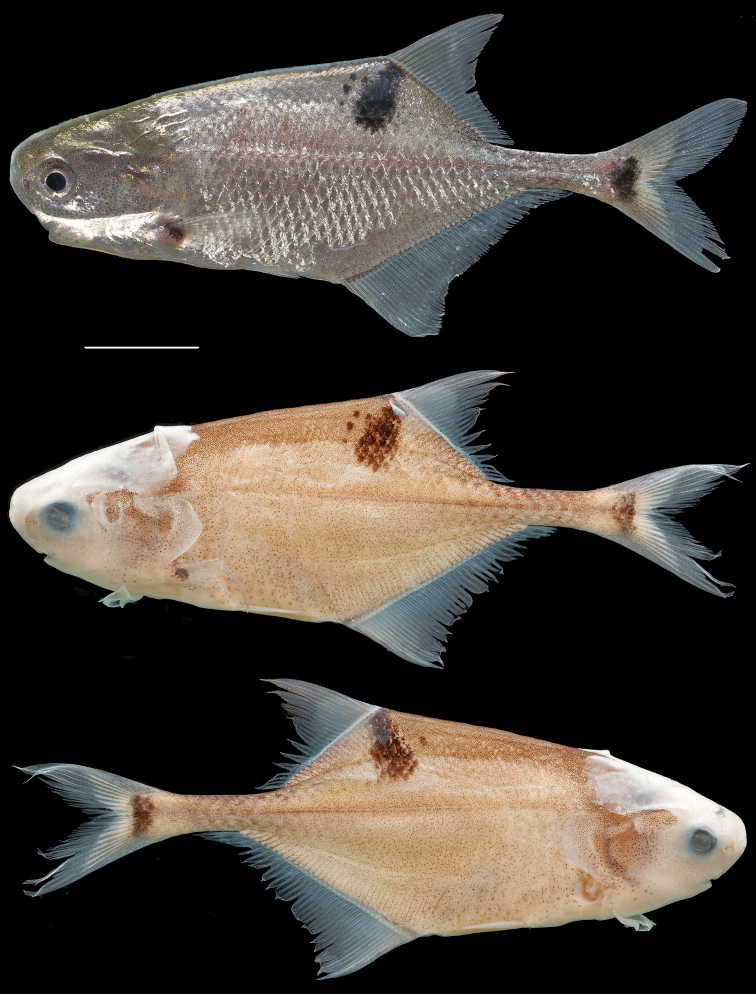
Photographs of the holotype of *Petrocephalus boboto* sp. n. from Yangambi, Congo River, Democratic Republic of Congo. Top photograph, the holotype specimen (56.9 mm standard length) before preservation showing appearance in life (CUMV 96774); middle and bottom photographs represent the left and right sides of the preserved holotype. Scale bar equals one centimeter.

Figs 2 and 3 ; Table 1

#### Holotype.

CUMV 96774, tag no. JPS-10-426, 56.9 mm SL, sex undetermined, Orientale Province, Democratic Republic of Congo: Congo River at Yangambi, 0.76°N, 24.24°E, Lavoué et al., 10 September 2010.

#### Diagnosis.

*Petrocephalus boboto* sp. n. is distinguished from all other *Petrocephalus* species of Central Africa (i.e., Lower Guinea and Congo provinces) by the following combination of characteristics: three distinctive bilateral black spots on body, one at origin of the pectoral fin, one at origin of caudal fin and one on flank just below anterior part of dorsal fin; two distinct electoreceptive rosettes in head (i.e., Nakenrosette and Khelrosette), the third rosette (Augenrosette) reduced in size to few knollenorgan pores; 23 branched rays in dorsal fin and 34 branched rays in anal fin; triphasic EOD waveform of normal polarity (i.e. first phase head-positive).

#### Description.

Morphometric ratios and meristic data for holotype presented in Table 1. Standard length = 56.9 mm. Sex undetermined: specimen lacks anal fin “notch” present in mature males, but may be juvenile. Body ovoid, 2.7 times longer than high and laterally compressed. Head length 3.3 times in standard length. Snout short (HL/SNL = 6.6) and round. Mouth small (HL/MW = 4.4), subterminal, opening under anterior half of eye. Teeth small and bicuspid, 13 in single row in upper jaw, 24 in single row in lower jaw. Nostrils closely set (distance between nostrils = 0.4 mm) with the posterior one very close to eye (distance between posterior nostril and eye = 0.1 mm). Dorsal and anal fins originating in posterior half of body (SL/PDD = 1.6 and SL/PAD = 1.8). Pre-dorsal distance slightly greater than pre-anal distance (PDD/PAD = 1.1). Dorsal fin with 23 branched rays. Anal fin with 34 branched rays. Pectoral fins with 9 rays. Pelvic fin with 6 rays. Distance between pelvic fin and anal fin = 10.1 mm. Distance between pectoral fin and anal fin = 16.4 mm. Scales cover body, except for head. Lateral line visible and complete with 34 pored scales along its length. Ten scales around caudal peduncle. Ten scales between anterior base of anal fin and lateral line. Caudal peduncle narrow (CPL/CPD = 1.6). Thick skin on head. Knollenorgans visible, clustered into two distinct rosettes, Nackenrosette and Kehlrosettes as described in Harder (1968). Knollenorgan pores in front of eye not highly condensed in the form of a discernable Augenrosette.

**Table 1.** Principal morphometric ratios and meristic counts for the holotype (CUMV 96774) of *Petrocephalus boboto* sp. n. from Yangambi, the holotype (MRAC 15191) of *Petrocephalus binotatus* from Ikengo, the holotype (CUMV 88074) and the 17 paratypes (CUMV 88076, 88079, 88041, 87838, 88063, 87785, 88052, 88053, 92390, 87830, 88080, 88123, 88064, 88065, 88031 and 88032) of *Petrocephalus arnegardi* sp. n., all from Odzala-Kokua National Park, and two other specimens (BMNH 2013.8.29.34 and 2013.8.29.125) of *Petrocephalus arnegardi* sp. n. from Yangambi. Abbreviations: u = sex undetermined; m = sexually mature male; Min-Max = minimum-maximum; stdev = standard deviation. “*” indicates data from Pellegrin (1924).

	*Petrocephalus boboto* sp. n.	*Petrocephalus binotatus*	*Petrocephalus arnegardi* sp. n.			
	Holotype (u) from Yangambi	Holotype (m) from Ikengo	Holotype (m) from Odzala	Paratypes from Odzala		Two non-types (u, m) from Yangambi
				Min–Max	Mean (stdev)	
Standard length (mm)	56.9	83.2	72.6	62.8–90.1	72.1	48.9, 76.0
Head length (mm)	17.4	23.1	21.6	17.3–24.6	20.3	12.3, 20.2
**Ratio of standard length (SL):**						
SL/body height (H)	2.7	2.4	2.5	2.3–2.8	2.6 (0.2)	2.8, 2.6
SL/head length (HL)	3.3	3.6	3.4	3.4–3.9	3.6 (0.2)	4.0, 3.8
SL/pre-dorsal distance (PDD)	1.6	1.6	1.5	1.4–1.6	1.5 (0.0)	1.6, 1.6
SL/pre-anal distance (PAD)	1.8	1.7	1.6	1.6–1.7	1.7 (0.0)	1.8, 1.7
SL/dorsal fin length (DFL)	5.1	4.5	4.9	4.2–5.4	5.0 (0.3)	5.0, 4.9
SL/anal fin length (AFL)	3.0	2.8	3.1	2.8–3.3	3.0 (0.2)	3.2, 3.1
SL/caudal peduncle length (CPL)	6.3	7.0	6.4	5.7–7.5	6.7 (0.5)	5.8, 7.6
SL/mouth width (MW)	14.2	11.6	15.1	14.7–18.4	16.4 (1.1)	18.0, 15.8
**Ratio of head length (HL):**						
HL/snout length (SNL)	6.6	5.5	8.3	6.5–9.3	7.5 (0.8)	5.1, 5.8
HL/mouth width (MW)	4.4	3.2	4.5	4.1–5.0	4.6 (0.2)	4.5, 4.2
HL/eye diameter (ED)	4.4	3.6	4.1	3.5–4.4	4.0 (0.3)	4.2, 4.7
HL/interorbital width (IOW)	3.7	2.3	3.3	2.6–3.9	3.2 (0.3)	2.9, 2.6
HL/head width (HW)	2.4	1.9	2.2	1.8–2.2	2.1 (0.1)	1.9, 1.9
HL/mouth position (MP)	4.4	3.9	5.5	4.2–5.6	4.8 (0.4)	4.4, 4.4
**Ratio of caudal peduncle length (CPL):**						
CPL/caudal peduncle depth (CPD)	1.6	2.3	2.2	1.8–2.5	2.2 (0.2)	2.6, 2.0
**Meristic counts:**						
Dorsal fin branched rays (DR)	23	24	20	20–22	21 (1)	20, 22
Anal fin branched rays (AR)	34	33	32	31–34	32 (1)	30, 32
Number of lateral line scales (SLL)	34	37	37	34–38	36 (1)	36, 36
Number of scale rows between anterior base of anal fin and lateral line (SDL)	10	11	12	10–13	11 (1)	10, 10
Number of teeth in upper jaw (TUJ)	13	15*	8	9–16	10 (1)	9, 11
Number of teeth in lower jaw (TLJ)	24	24*	20	20–24	21 (1)	25, 21

**Electric organ discharge** (Fig. 2A). Short triphasic EOD waveform, first phase head-positive. Relative height of peak 1 = 0.241, relative height of peak 2 = -0.759, relative height of peak 3 = 0.053, duration of peak 1 = 0.137 msec, duration of peak 2 = 0.041 msec, duration of peak 3 = 0.079 msec, total EOD duration = 0.257 msec. Based on characteristics of the EOD, the electrocytes are assumed to have non-penetrating stalks and to be innervated posteriorly (Sullivan et al. 2000).

**Live coloration** (Fig. 3). Body background color uniformly gray/silver with metallic reflection on the flanks and head. Light melanophores densely and evenly distributed on body, slightly larger on head. Three distinct black marks on each side of the body, one at the base of the pectoral fins, one at the base of the caudal fin and one subdorsal, below the first anterior rays of the dorsal fin. All fins hyaline.

**Preserved coloration** (Fig. 3). Background beige with dorsum slightly darker. Numerous light melanophores visible all over body. Thick skin on head and fins opaque. Three distinct melanin marks on side of body distinctly visible.

#### Distribution

(Fig. 1). Holotype (and only specimen known) of *Petrocephalus boboto* sp. n. collected from the main channel of the Congo River, at Yangambi, Orientale Province, Democratic Republic of Congo.

#### Phylogenetic affinities

**Figure 4.**
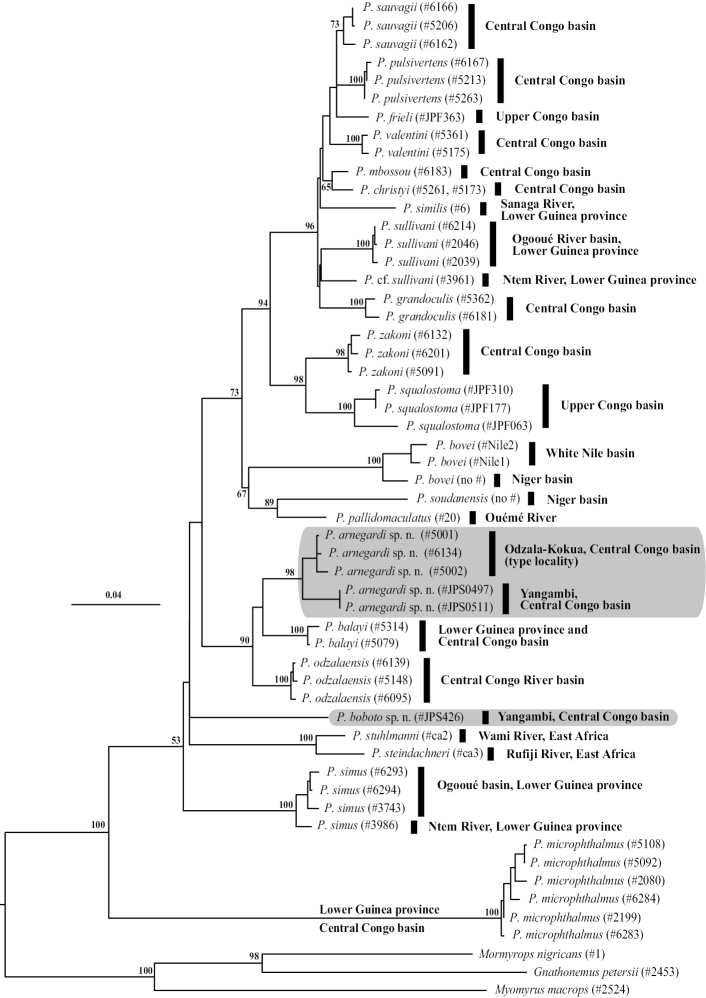
Phylogeny of *Petrocephalus* (23 species, 52 specimens) estimated by maximum likelihood analysis of cytochrome *b* nucleotide sequences. Mormyrin *Gnathonemus petersii*, *Mormyrops nigricans* and *Myomyrus macrops* were used as outgroups to root the tree. Numbers at internal branches are bootstrap proportions (in %) shown only for interspecific relationships and when they exceed 50%. Black-filled vertical bars to the right of the tree indicate the river basin origins of the specimens. The scale bar corresponds to 0.04 substitutions per site. *Petrocephalus boboto* sp. n. and *Petrocephalus arnegardi* sp. n. are highlighted in gray.

(Fig. 4). *Petrocephalus boboto* sp. n. represents a deep lineage within the genus *Petrocephalus* not closely related to any of the four described species exhibiting a similar pattern of markings: *Petrocephalus zakoni*, *Petrocephalus odzalaensis*, *Petrocephalus balayi* and *Petrocephalus arnegardi* sp. n. However, it is worth noting that *Petrocephalus binotatus*, the fifth species with a potentially similar pigmentation, is not included in this tree and the phylogenetic position of this species is unresolved.

#### Etymology.

The name *boboto* is a word in Lingala, the language spoken at the type locality of *Petrocephalus boboto* sp. n., meaning “peace,” alluding to the right of all people of D.R. Congo to live in peace and safety.

#### Comparison.

The distinctive pattern of melanin markings of *Petrocephalus boboto* sp. n., having three dark spots—one at the origin of the pectoral fin, a second at the origin of the caudal fin, and a third on the flank just below the anterior base of the dorsal fin—distinguishes this species from most of its congeners. Only five other species of *Petrocephalus* similarly possess three black marks in these locations: *Petrocephalus odzalaensis*, *Petrocephalus balayi*, *Petrocephalus zakoni*, *Petrocephalus binotatus* and *Petrocephalus arnegardi* sp. n. (the comparison with *Petrocephalus arnegardi* sp. n. is provided under the description of this species). *Petrocephalus boboto* sp. n. can immediately be distinguished from *Petrocephalus zakoni* by the presence of two of the three electroreceptor rosettes on the head that are typical to *Petrocephalus* (versus absence of all three in *Petrocephalus zakoni*) and a higher number of anal fin rays (33 in *Petrocephalus boboto* sp. n. versus a maximum of 28 in *Petrocephalus zakoni*). *Petrocephalus boboto* sp. n. can be distinguished from *Petrocephalus balayi* and *Petrocephalus odzalaensis* by its higher number of anal fin rays (33 in *Petrocephalus boboto* sp. n. versus a maximum of 27 and 20 in *Petrocephalus balayi* and *Petrocephalus odzalaensis*, respectively). *Petrocephalus balayi* also has a proportionally larger mouth (HL/MW = 2.7–3.9, mean = 3.3 versus 4.4 in *Petrocephalus boboto* sp. n.). *Petrocephalus odzalaensis* has a proportionally wider head (HL/IOW = 2.5–3.0, mean = 2.8 and HL/HW = 1.7–1.8, mean = 1.7 versus 3.7 and 2.7 in *Petrocephalus boboto* sp. n., respectively) and its body background color is more pinkish rather than gray/silver in *Petrocephalus boboto* sp. n. *Petrocephalus boboto* sp. n. is distinguished from the holotype of *Petrocephalus binotatus* by a smaller mouth (SL/MW = 14.2 and HL/MW = 4.4 versus 11.6 and 3.2, respectively, in the holotype of *Petrocephalus binotatus*), a smaller eye (HL/ED = 4.4 versus 3.6 in the holotype of *Petrocephalus binotatus*), and a smaller interorbital distance (HL/IOW = 3.7 versus 2.3 in the holotype of *Petrocephalus binotatus*).

The holotype of *Petrocephalus boboto* sp. n. is smaller than the holotype of *Petrocephalus binotatus*. The differences between *Petrocephalus boboto* sp. n. and *Petrocephalus binotatus* are unlikely to be the result of growth allometry since allometric differences have only been observed with respect to the number of teeth in some species of *Petrocephalus* (Bigorne and Paugy 1991), a character that does not differentiate these two species. Although we are confident of the specific distinction between *Petrocephalus boboto* sp. n. and *Petrocephalus binotatus*, more specimens are needed to better characterize the morphological, molecular and electrophysiological differences between them.

### Petrocephalus arnegardi sp. n.

http://zoobank.org/23BDE2CA-426C-469F-B124-07E73C22EE82

http://species-id.net/wiki/Petrocephalus_arnegardi

**Figure 5.**
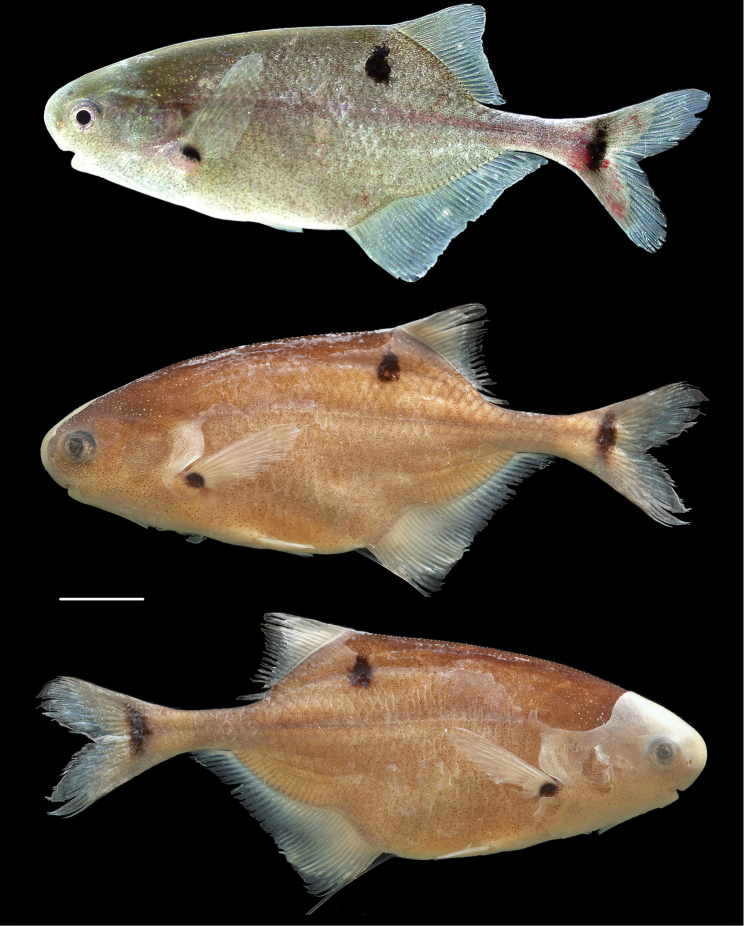
Photographs of type specimens of *Petrocephalus arnegardi* sp. n. from Odzala-Kokua National Park, Congo River, Republic of the Congo. Top photograph, a paratype specimen (CUMV 92390, 72.0 mm standard length) before preservation showing appearance in life; middle and bottom photographs represent the left and right sides of the preserved holotype (CUMV 88074, 71.6 mm standard length). Scale bar equals one centimeter.

Figs 2 and 5 ; Table 1

Petrocephalus sp. 1, OTU 1 in Lavoué et al. (2008).
Petrocephalus binotatus in Lavoué et al. (2010), Lavoué (2011, 2012), Carlson et al. (2011).

#### Holotype.

CUMV 88074, tag no. 5074, 72.6 mm SL, male, Republic of the Congo: Cuvette Ouest, Congo River basin, Pandaka River, Odzala-Kokua National Park, 0.62°N, 14.92°E, Friel et al., August 2002.

#### Paratypes

**(17).** Republic of the Congo: Cuvette-Ouest: Congo River basin: CUMV 88076, tag no. 5076, 69.6 mm SL, male, same data as holotype; CUMV 88041, tag no. 5120, 85.1 mm SL, sex undetermined, same data as holotype; CUMV 88080, tag no. 5083, 72.0 mm SL, male, same data as holotype; CUMV 88032, tag no. 5101, 73.0 mm SL, male, same data as holotype; CUMV 87785, tag no. 5097, 71.3 mm SL, sex undetermined, same data as holotype; CUMV 88031, tag no. 5100, 73.7 mm SL, sex undetermined, same data as holotype; CUMV 88079, tag no. 5082, 74.8 mm SL, sex undetermined, Lékénie River at Mboko landing, Odzala-Kokua National Park, 0.62°N, 14.90°E, Friel et al., August 2002; CUMV 88063, tag no. 5197, 64.9 mm SL, sex undetermined, Lékénie River at Mboko landing, Odzala-Kokua National Park, 0.62°N, 14.90°E, Friel et al., August 2002; CUMV 88065, tag no. 5002, 70.7 mm SL, sex undetermined [cytochrome *b* gene determined], Lékénie River at Mboko landing, Odzala-Kokua National Park, 0.62°N, 14.90°E, Friel et al., August 2002; CUMV 88064, tag no. 5001, 63.9 mm SL, male [cytochrome *b* gene determined], Lékénie River at Mboko landing, Odzala-Kokua National Park, 0.62°N, 14.90°E, Friel et al., August 2002; CUMV 88052, tag no. 5158, 75.1 mm SL, male, Lékénie River at Mboko landing, Odzala-Kokua National Park, 0.62°N, 14.90°E, Friel et al., August 2002; CUMV 88053, tag no. 5159, 69.3 mm SL, male, Lékénie River at Mboko landing, Odzala-Kokua National Park, 0.62°N, 14.90°E, Friel et al., August 2002; CUMV 88123, tag no. 5377, 68.9 mm SL, male, Lékoli River, Odzala-Kokua National Park, 0.61°N, 14.93°E, Friel et al., August 2002; CUMV 87838, tag no. 5404, 90.1 mm SL, male, Lokoué River, Odzala-Kokua National Park, 0.90°N, 15.12°E, Friel et al., August 2002; CUMV 87830, tag no. 5395, 72.3 mm SL, sex undetermined, Lokoué River, Odzala-Kokua National Park, 0.90°N, 15.12°E, Friel et al., August 2002; CUMV 92390, two specimens, tag no. 6133, 62.8 mm SL, male [cytochrome *b* gene determined] and tag no. 6134, 67.5 mm SL, male [cytochrome *b* gene determined], mouth of the Lékéni River near the Lékoli River, Odzala-Kokua National Park, 0.62°N, 14.91°E, Arnegard et al., June 2006.

#### Other specimens

**(18).** Republic of the Congo: Cuvette-Ouest: Congo River basin: Lékénie River at Mboko landing, Odzala-Kokua National Park, 0.62°N, 14.90°E, Friel et al., August 2002: CUMV 88066, tag no. 5028, 59.0 mm SL, sex undetermined; CUMV 88046, tag no. 5126, SL not measured, sex undetermined; Republic of the Congo: Cuvette-Ouest: Congo River basin: Pandaka River, Odzala-Kokua National Park, 0.62°N, 14.92°E, Friel et al., August 2002: CUMV 88075, tag no. 5075, 71.0 mm SL, sex undetermined; CUMV 88081, tag no. 5084, 73.0 mm SL, male; CUMV 88082, tag no. 5085, 54.0 mm SL, sex undetermined; CUMV 88028, tag no. 5096, 74.0 mm SL, male; CUMV 88029, tag no. 5098, 74.0 mm SL, male; CUMV 88043, tag no. 5122, 73.0 mm SL, male; CUMV 88044, tag no. 5123, 66.0 mm SL, male; CUMV 88045, tag no. 5124, 73.0 mm SL, male; Republic of the Congo: Cuvette-Ouest: Congo River basin: Lokoué River, Odzala-Kokua National Park, 0.90°N, 15.12°E, Friel et al., August 2002: CUMV 88125, tag no. 5396, 74.0 mm SL, male; Republic of the Congo: Cuvette-Ouest: Congo River basin: small channel around island in Lékoli River, Odzala-Kokua National Park, 0.62°N, 14.92°E, Friel et al., August 2002: CUMV 88107, tag no. 5276, SL not measured, male; Republic of the Congo: Cuvette-Ouest: Congo River basin: Lékoli River, Odzala-Kokua National Park, 0.61°N, 14.93°E, Friel et al., August 2002: CUMV 88067, tag no. 5029, 59.0 mm SL, sex undetermined; CUMV 88068, tag no. 5030, 59.0 mm SL, sex undetermined; CUMV 88069, tag no. 5031, 57.0 mm SL, sex undetermined; Republic of the Congo: Cuvette-Ouest: Congo River basin: small stream entering Mambili River from the east between Moba and Lokoué, 0.87°N, 15.11°E, Friel et al. August 2002: CUMV 88128, tag no. 5423, 70.0 mm SL, male; Democratic Republic of Congo: Orientale Province: Congo basin: BMNH 2013.8.29.34, tag no. JPS-497, 76.0 mm SL, male [cytochrome *b* gene determined, no EOD recorded], Lifundu River, 5 km downstream of Yangambi, 0.76°N, 24.24°E, Lavoué & Thumitho, 11 September 2010; BMNH 2013.8.29.125, tag no. JPS-511, 48.9 mm SL, sex undetermined [cytochrome *b* gene determined], Congo River at Yangambi, 0.76°N, 24.24°E, Lavoué et al., 11 September 2010.

#### Diagnosis.

*Petrocephalus arnegardi* sp. n. is distinguished from all other *Petrocephalus* species of Central Africa by the following combination of characteristics. Pigmentation pattern comprising three well-defined, bilateral black patches: one usually distinct (sometimes reduced in size, but rarely absent) round/ovoid subdorsal black mark situated slightly anterior to dorsal, one black mark at the base of each pectoral fin, and one ovoid black mark centered at the base of caudal fin. Dorsal fin at least one third shorter than anal fin (AFL/DFL ≥ 1.5, range = 1.5–1.7). Dorsal fin with at least 20 branched rays but no more than 22. Anal fin with at least 30 branched rays (range = 30–34). Sixteen teeth or fewer (range = 8–16) in upper jaw, 25 teeth or fewer (range = 20–25) in lower jaw. Eye relatively large (HL/ED ≤ 4.7, range = 3.5–4.7). Mouth subterminal; ratio of head length to mouth position (HL/MP) between 4.2 and 5.6. Mouth small (HL/MW ≥ 4.1, range = 4.1–5.0). EOD of normal polarity, mainly biphasic with sometimes the presence of a small-amplitude positive third phase.

#### Description.

This description is based on the material from Odzala-Kokua National Park in the Republic of the Congo. Morphometric ratios and meristic data for the holotype and 17 paratypes are presented in Table 1. Maximum SL observed = 90.1 mm, holotype = 72.6 mm). Body ovoid, longer than high (2.3 ≤ SL/H ≤ 2.8, average = 2.6, holotype = 2.5) and laterally compressed. Head length between 3.4 and 4.0 times in standard length (average = 3.6, holotype = 3.4). Snout short (6.5 ≤ HL/SNL ≤ 9.3, average = 7.5, holotype = 8.3) and round. Eye large (3.5 ≤ HL/ED ≤ 4.4, average = 4.0, holotype = 4.1). Mouth small (4.1 ≤ HL/MW ≤ 5.0, average = 4.6, holotype = 4.5), subterminal, opening under the anterior half of the eye. Teeth small and bicuspid, 8 to 16 (median = 10) in a single row in the upper jaw, 20 to 24 (median = 21) in the lower jaw. Dorsal and anal fins originate in the posterior half of the body (1.4 ≤ SL/PDD ≤ 1.6 and 1.6 ≤ SL/PAD ≤ 1.7, respectively). Pre-dorsal distance slightly greater than the pre-anal distance (PDD/PAD = 1.1). Dorsal fin with 20–22 branched rays (median = 21). Anal fin with 31–34 branched rays (median = 32, holotype = 32). Scales cover the body, except for the head. Lateral line visible and complete with 34 to 38 pored scales along its length. Ten to 13 scales (median = 11), between the anterior base of the anal fin and the lateral line. Caudal peduncle thin (1.8 ≤ CPL/CPD ≤ 2.5, average = 2.2, holotype = 2.2). Twelve scales around the caudal peduncle. Skin on head thick. The three rosettes of Knollenorgans, Augenrosette, Nackenrosette and Kehlrosette, are present on the head.

**Electric organ discharge.** Statistics for waveform landmarks and other EOD measurements are provided by Lavoué et al. (2008) for specimens recorded in Odzala-Kokua National Park, including the holotype and paratypes (specimens listed in Suppl. material 1). *Petrocephalus arnegardi* sp. n. produces EOD waveforms largely similar to those of many species of this genus. In Odzala-Kokua, mean EOD duration (± std. dev.) is 0.330 ± 0.074 msec in sexually mature males and 0.270 ± 0.033 msec in other sex undetermined specimens. The EOD waveform characteristics of the only recorded specimen of *Petrocephalus arnegardi* sp. n. of Yangambi (Fig. 2B; EOD biphasic, relative height of peak 1 = 0.216, relative height of peak 2 = -0.784, duration of peak 1 = 0.185 msec, duration of peak 2 = 0.075 msec, total EOD duration = 0.260 msec) are similar to those of Odzala-Kokua specimens in all respects. Based on characteristics of the EODs, the electrocytes are assumed to have non-penetrating stalks and to be innervated posteriorly (Sullivan et al. 2000).

**Live coloration** (Fig. 5; see also Fig. 3 in Lavoué et al. 2010). Body uniformly silvery white, with three distinct bilateral melanin marks: a distinct, ovoid black mark situated slightly anterior to the dorsal fin, sometimes covering only a few scales, a black spot at the base of the pectoral fin and a somewhat vertically oriented ovoid black mark centered at the base of the caudal fin that does not extend onto the upper and lower parts of the caudal fin. Fins hyaline.

**Preserved coloration** (Fig. 5; see also Fig. 3 in Lavoué et al. 2010). Body light brown, with head and dorsum slightly darker. Thick skin on head opaque. Numerous light melanophores on body, slightly larger ventrally from the snout to the anal fin. Fins whitish/opaque.

**Distribution** (Fig. 1). Endemic to the Congo River basin. Holotype and paratypes from Odzala-Kokua National Park (Republic of the Congo) where they were mainly collected along the main channel of the Lékoli River, northwestern Congo River basin. The two specimens collected at Yangambi will extend the distribution to the eastern part of the Congo basin’s central cuvette. Abundant in Odzala-Kokua but apparently rare at Yangambi (Poll and Gosse 1963).

**Phylogenetic affinities** (Fig. 4). The Yangambi specimens and the Odzala-Kokua specimens of *Petrocephalus arnegardi* sp. n. are sister groups in the cytochrome *b* phylogenetic tree. *Petrocephalus arnegardi* sp. n. belongs to a clade containing *Petrocephalus odzalaensis* and *Petrocephalus balayi*, two other species with a similar melanin pattern composed of three distinct black marks, but it is only distantly related to *Petrocephalus boboto* sp. n. As previously noted, *Petrocephalus binotatus* is absent in this tree and its phylogenetic position is unknown.

#### Etymology.

This species is dedicated to Matthew E. Arnegard, our friend and colleague, in recognition of his contributions to study of mormyrid evolution and diversification (e.g., Arnegard et al. 2005; Arnegard and Carlson 2005; Arnegard et al. 2010a; Arnegard et al. 2010b). Matthew Arnegard is additionally a member of the “*Mintotom* Team”: researchers associated with the Carl D. Hopkins Laboratory at Cornell University who have conducted field studies on African weakly electric fishes for more than 15 years. (“*Mintotom*” is the plural form of the word for mormyrid fish in the Fang language of West Central Africa.).

#### Comparisons.

**Figure 6.**
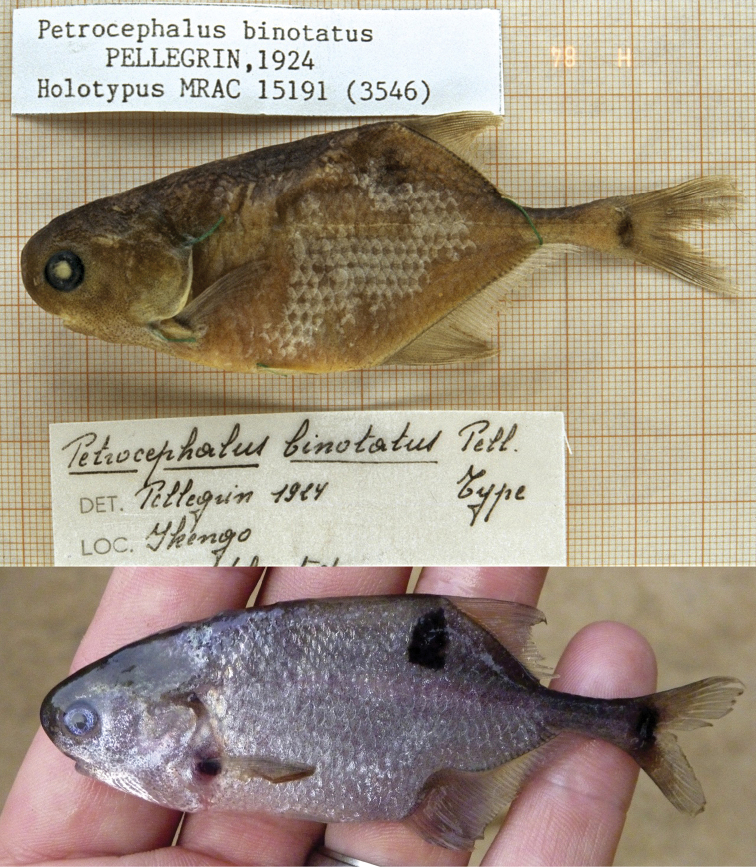
Top photograph, the preserved holotype of *Petrocephalus binotatus* (MRAC 15191; 83.2 mm standard length) collected at Ikengo near the modern locality of Mbandaka, Equateur Province, in the Central Congo basin of Democratic Republic of Congo (Photo by Wilhelm Harder, reproduced courtesy of ETI Bioinformatics); bottom photograph, a specimen of *Petrocephalus arnegardi* (BMNH 2013.8.29.34) before preservation showing appearance in life, from Yangambi, Congo River, Democratic Republic of Congo.

As for *Petrocephalus boboto* sp. n., the presence of three dark spots in *Petrocephalus arnegardi* sp. n. distinguishes this species from most of its congeners. As for other species having a similar pattern of melanin marking, *Petrocephalus arnegardi* sp. n. can easily be distinguished from *Petrocephalus zakoni* by the presence of three electroreceptor rosettes on the head (versus absence of all three in *Petrocephalus zakoni*) and a higher number of anal fin rays (a minimum of 30 in *Petrocephalus arnegardi* sp. n. versus a maximum of 28 in *Petrocephalus zakoni*). Its high number of anal fin rays distinguishes *Petrocephalus arnegardi* sp. n. from *Petrocephalus balayi* and *Petrocephalus odzalaensis* (30–34 in *Petrocephalus arnegardi* sp. n. versus a maximum of 27 and 20 in *Petrocephalus balayi* and *Petrocephalus odzalaensis*, respectively). *Petrocephalus balayi* has a proportionally larger mouth (HL/MW = 2.7–3.9 versus 4.1–5.0 in *Petrocephalus arnegardi* sp. n.). *Petrocephalus arnegardi* sp. n. generally resembles to the holotype of *Petrocephalus binotatus*, leading Lavoué et al. (2010) to assign these specimens from the Odzala-Kokua National Park to *Petrocephalus binotatus*. However, *Petrocephalus arnegardi* sp. n. has a distinctly smaller mouth than *Petrocephalus binotatus* (HL/MW = 4.4–5.2 in Odzala-Kokua specimens and 4.2 and 4.5 in the two Yangambi specimens *versus* 3.2 in the holotype of *Petrocephalus binotatus*) and a smaller interorbital width (HL/IOW ≥ 2.6 in Odzala-Kokua and Yangambi specimens *versus* 2.3 in the holotype of *Petrocephalus binotatus*). The faded pigmentation in the preserved holotype of *Petrocephalus binotatus* precludes its accurate description and comparison (Fig. 6). Whereas a faded roundish black mark situated slightly anterior to the dorsal fin on each side of the flank and an ovoid black mark centered at the base of the caudal fin are visible on the preserved holotype, the presence of a black mark at the base of the pectoral fin is ambiguous (Fig. 6). In his description of *Petrocephalus binotatus*, Pellegrin (1924) mentioned the subdorsal mark and the mark at the base of the anal fin, but did not make reference to any black mark at the origin of the pectoral fin. The black mark at the base of the pectoral fin in *Petrocephalus arnegardi* sp. n. is always present and intense. Pellegrin also described the black mark at the base of the caudal fin as crescent-like, extending onto the upper and lower rays of this fin (see drawing in Pellegrin 1928) whereas on the holotype this mark appears more ovoid and does not seem to extend onto any fin rays (Fig. 6). *Petrocephalus arnegardi* sp. n. is distinguished from *Petrocephalus boboto* sp. n. by a distinctly smaller mouth (SL/MW ≥ 14.7, range = 14.7–18.4 versus 14.2 in *Petrocephalus boboto* sp. n.), a slightly larger interorbital distance (HL/IOW ≤ 3.9, mean = 3.2 versus 3.7 in *Petrocephalus boboto* sp. n.) and the presence of a well-defined Augenrosette (versus reduced in *Petrocephalus boboto* sp. n.). In our phylogenetic tree (Fig. 4), *Petrocephalus boboto* sp. n. is not the sister group of *Petrocephalus arnegardi* sp. n.

### Key to the *Petrocephalus* species of the Central Congo basin

**Table:** 

1	Dorsal fin with fewer than 18 branched rays (rarely 18); only eight to 10 scale rows between the anterior base of the anal fin and the lateral line; distinct melanin markings absent on body (i.e., absence of black patches that are species-specific for many other *Petrocephalus* species); rosettes of Knollenorgan electroreceptors absent on the head	*Petrocephalus microphthalmus* group
	Note: Currently, the *Petrocephalus microphthalmus* group contains three morphologically similar valid species: *Petrocephalus microphthalmus*, *Petrocephalus haullevillii*, and *Petrocephalus schoutedeni*. The identification of each of these three species is currently challenging pending their taxonomic revision.
–	Dorsal fin usually with more than 20 branched rays (sometimes 20; in very rare instances 19); at least 10, usually more, scale rows between the anterior base of the anal fin and the lateral line; distinct melanin markings (black patches) may be present or absent on the body; electroreceptor rosettes present or absent on the head	2
2	Mouth large, its width at most 3.9 times in head length; at least 15 teeth in upper jaw, usually more	3
–	Mouth small, its width at least 3.6 times (usually 4.0–4.4 times) in head length; usually fewer than 15 teeth in upper jaw (rarely 15 or 16)	5
3	Anal fin contains 26 or 27 branched rays; 20–22 branched rays in the dorsal fin; three bilateral intense black patches present: a rounded sub-dorsal mark, an ovoid caudal mark and a mark at the origin of the pectoral fin	*Petrocephalus balayi*
–	Anal fin with more than 30 branched rays; more than 24 branched rays in dorsal fin; only two distinct, bilateral black patches: a sub-dorsal mark and a caudal mark	4
4	Eye relatively small (HL/ED ≥ 4.0); mouth subterminal (HL/MP ≥ 4.4), opening under the anterior half of the eye; two distinct melanin marks present but sometimes pale: a rounded sub-dorsal mark and a crescent-like mark at the base of the caudal fin; Knollenorgan electroreceptors organized into three distinct rosettes on head, but rosettes relatively small; EOD waveform typical for the genus, polarity normal (see Fig. 10C in Lavoué et al. 2010)	*Petrocephalus sauvagii*
–	Eye large (HL/ED ≤ 3.5); mouth subterminal but positioned more posteriorly along the ventral margin of the head (HL/MP ≤ 3.5), opening under the posterior half of the eye; two distinct melanin marks: a rounded, sometimes irregularly shaped, sub–dorsal black mark and a crescent-like black mark at the base of the caudal fin; three larger rosettes of Knollenorgan electroreceptors present on the head; EOD waveform very distinctive among congeners, appearing to be reversed in polarity compared to EODs of all other *Petrocephalus* species (see Fig. 11C in Lavoué et al. 2010)	*Petrocephalus pulsivertens*
5	Anal fin contains 30 or more branched rays; melanin markings (black patches) present on the body and always distinctly visible	6
–	Anal fin contains at most 29 branched rays (usually fewer); melanin markings present but sometimes hardly visible	9
6	Dorsal fin contains 24–26 branched rays; eye large (HL/ED ≤ 3.2); mouth very small relative to many congeners (HL/MW ≥ 5.2); two melanin marks present and distinct but of medium intensity: a rounded sub-dorsal mark and a crescent-like mark at the base of the caudal fin; two readily observable rosettes of Knollenorgan electroreceptors present on the head (Augenrosette and Nackenrosette) plus a Kehlrosette that is rather difficult to observe without staining	*Petrocephalus grandoculis*
–	Dorsal fin contains 24 or fewer branched rays; eye smaller in size (3.5 ≤ HL/ED ≤ 4.7); mouth larger (HL/MW ≤ 5.2); three distinct melanin marks (black patches) present: an ovoid sub-dorsal mark (sometimes small but easily discernable), an ovoid mark at the base of the caudal fin and a mark at the origin of the pectoral fin; Nackenrosette and Kehlrosette present on the head and distinct; Augenrosette present but sometimes reduced in size to few, not densely packed, Knollenorgan pores	7
7	Augenrosette present but reduced in size to few Knollenorgan pores; narrow head (HL/HW = 2.4 and HL/IOW = 3.7)	*Petrocephalus boboto* sp. n.
–	Augenrosette present and well distinct; wider head (HL/HW ≤ 2.2 and HL/IOW ≤ 3.9, average = 3.2)	8
8	Large mouth (SL/MW = 11.6 and HL/MW = 3.2); interorbital distance large (HL/IOW = 2.3); 24 dorsal fin branched rays	*Petrocephalus binotatus*
–	Smaller mouth (SL/MW ≥ 14.7 and HL/MW ≥ 4.1); shorter interorbital distance (HL/IOW ≥ 2.6); 22 or fewer dorsal fin branched rays	*Petrocephalus arnegardi* sp. n.
9	Melanin markings on body intensely black with sharply defined edges, forming characteristic shapes (e.g., very rounded black sub-dorsal spot or saddle-like sub-dorsal patch, crescent shaped black mark at the base of the caudal fin or round black spot at the caudal fin base)	10
–	Melanin markings of much weaker intensity, consisting of more irregularly–shaped patches and with diffuse edges	12
10	Small but intense black mark present on each side of the body at the pectoral fin origin; Knollenorgan electroreceptors on the head may or may not be arranged into discrete clusters (i.e., rosettes may be present or absent), but if present, Augenrosette is always as well developed as other two rosettes	11
–	No distinct black mark visible at the origin of the pectoral fin; electroreceptors organized into three distinct rosettes on the head, but the Augenrosette is small and not as well developed as other two rosettes	*Petrocephalus christyi*
11	Eye large (HL/ED ≤ 3.3); sub-dorsal black patch often contacting contralateral mark over dorsum and anteriormost branched rays of the dorsal fin; caudal melanin mark forming a rather uniformly shaped crescent (or “V”) extending onto upper and lower fleshy lobes of caudal fin; Knollenorgan electroreceptors on the head not clustered into discrete groups (i.e., rosettes absent)	*Petrocephalus zakoni*
–	Eye small (HL/ED ≥ 3.7); sub-dorsal black patch distinctly rounded, never in contact with contralateral mark and not extending onto dorsal fin; caudal mark ovoid rather than crescent- or V-shaped, not extending onto upper and lower parts of caudal fin; Knollenorgans on the head clustered into three rosettes	*Petrocephalus odzalaensis*
12	Mouth subterminal, opening under the anterior half of the eye; snout short (HL/SNL ≥ 6.5); Knollenorgan electroreceptors on head are clustered into three rosettes (but a distinctive Kehlrosette is difficult to observe without staining); EOD of normal polarity, often appearing to have an overall biphasic waveform at low gain, although a minute third peak is in fact present (first head-positive peak, P1, much larger in amplitude than second head-positive peak, P3, which never exceeds 10% of total peak-to-peak amplitude)	*Petrocephalus valentini*
–	Mouth subterminal but positioned more caudally along ventral margin of head, opening under posterior half of eye; snout somewhat longer (HL/SNL = 5.4 in single specimen available, holotype); Knollenorgans on head clustered into only two rosettes (Nackenrosette and the Kehlrosette), Augenrosette absent; EOD of normal polarity, with more than two phases apparent even at low gain (the only specimen recorded exhibits an EOD containing 4 peaks; the second head-positive peak, P3, is larger in amplitude than the first head-positive peak, P1; amplitude of P3 substantially greater than 10% of total peak-to-peak amplitude)	*Petrocephalus mbossou*

## Supplementary Material

XML Treatment for Petrocephalus boboto

XML Treatment for Petrocephalus arnegardi

## Supplementary material

Supplementary material 1 List of specimens of *Petrocephalus arnegardi* and *Petrocephalus boboto* with EOD recording. doi: 10.3897/zookeys.400.6743.app1 Data type: species data Explanation note: List of specimens of *Petrocephalus arnegardi* and *Petrocephalus boboto* with electric organ discharge (EOD) recording (holotypes, paratypes, non types). Copyright notice: This dataset is made available under the Open Database License (http://opendatacommons.org/licenses/odbl/1.0/). The Open Database License (ODbL) is a license agreement intended to allow users to freely share, modify, and use this Dataset while maintaining this same freedom for others, provided that the original source and author(s) are credited.Sébastien Lavoué, John P. Sullivan
