# Supplementary material for: Petrocephalus boboto and Petrocephalus arnegardi, two new species of African electric fish (Osteoglossomorpha, Mormyridae) from the Congo River basin
Source: Zookeys. 2014 Apr 10;(400):43–65. doi: 10.3897/zookeys.400.6743 (PMC4023242; doi:10.3897/zookeys.400.6743)
Supplement: Supplementary material 1 — List of specimens of Petrocephalus arnegardi and Petrocephalus boboto with EOD recording. [file zookeys-400-043-s001.docx]

**Appendix I.** List of specimens of *Petrocephalus arnegardi* and *Petrocephalus boboto* with electric organ discharge (EOD) recording (holotypes, paratypes, non types); abbreviations: CUMV=Cornell University Museum of Vertebrates, CUML=Macaulay Library at the Cornell Lab of Ornithology, m=mature male, o=sex undetermined. EOD recording files available upon request to the [Macaulay Library](http://macaulaylibrary.org/order/information).

| **Specimens (N = 36)** | **Specimen tag** | **Specimen catalog no.** | **EOD catalog no.** | **Year** | **Sex** | **SL** |
| --- | --- | --- | --- | --- | --- | --- |
| *Petrocephalus arnegardi* (paratype) (Odzala) | 5001 | [CUMV 88064](http://arctos.database.museum/guid/CUMV:Fish:88064) | CUML 135561 | 2002 | m | 64 |
| *Petrocephalus arnegardi* (paratype) (Odzala) | 5002 | [CUMV 88065](http://arctos.database.museum/guid/CUMV:Fish:88065) | CUML 135562 | 2002 | o | 71 |
| *Petrocephalus arnegardi* (non type) (Odzala) | 5028 | [CUMV 88066](http://arctos.database.museum/guid/CUMV:Fish:88066) | CUML 135563 | 2002 | o | 59 |
| *Petrocephalus arnegardi* (non type) (Odzala) | 5029 | [CUMV 88067](http://arctos.database.museum/guid/CUMV:Fish:88067) | CUML 135564 | 2002 | o | 59 |
| *Petrocephalus arnegardi* (non type) (Odzala) | 5030 | [CUMV 88068](http://arctos.database.museum/guid/CUMV:Fish:88068) | CUML 135565 | 2002 | o | 59 |
| *Petrocephalus arnegardi* (non type) (Odzala) | 5031 | [CUMV 88069](http://arctos.database.museum/guid/CUMV:Fish:88069) | CUML 135566 | 2002 | o | 57 |
| *Petrocephalus arnegardi* (holotype) (Odzala) | 5074 | [CUMV 88074](http://arctos.database.museum/guid/CUMV:Fish:88074) | CUML 135567 | 2002 | m | 73 |
| *Petrocephalus arnegardi* (non type) (Odzala) | 5075 | [CUMV 88075](http://arctos.database.museum/guid/CUMV:Fish:88075) | CUML 135568 | 2002 | o | 71 |
| *Petrocephalus arnegardi* (paratype) (Odzala) | 5076 | [CUMV 88076](http://arctos.database.museum/guid/CUMV:Fish:88076) | CUML 135569 | 2002 | m | 70 |
| *Petrocephalus arnegardi* (paratype) (Odzala) | 5082 | [CUMV 88079](http://arctos.database.museum/guid/CUMV:Fish:88079) | CUML 135570 | 2002 | o | 75 |
| *Petrocephalus arnegardi* (paratype) (Odzala) | 5083 | [CUMV 88080](http://arctos.database.museum/guid/CUMV:Fish:88080) | CUML 135571 | 2002 | m | 72 |
| *Petrocephalus arnegardi* (non type) (Odzala) | 5084 | [CUMV 88081](http://arctos.database.museum/guid/CUMV:Fish:88081) | CUML 135572 | 2002 | m | 73 |
| *Petrocephalus arnegardi* (non type) (Odzala) | 5085 | [CUMV 88082](http://arctos.database.museum/guid/CUMV:Fish:88082) | CUML 135573 | 2002 | o | 54 |
| *Petrocephalus arnegardi* (non type) (Odzala) | 5096 | [CUMV 88028](http://arctos.database.museum/guid/CUMV:Fish:88028) | CUML 135574 | 2002 | m | 74 |
| *Petrocephalus arnegardi* (paratype) (Odzala) | 5097 | [CUMV 87785](http://arctos.database.museum/guid/CUMV:Fish:87785) | CUML 135575 | 2002 | o | 71 |
| *Petrocephalus arnegardi* (non type) (Odzala) | 5098 | [CUMV 88029](http://arctos.database.museum/guid/CUMV:Fish:88029) | CUML 135576 | 2002 | m | 74 |
| *Petrocephalus arnegardi* (paratype) (Odzala) | 5100 | [CUMV 88031](http://arctos.database.museum/guid/CUMV:Fish:88031) | CUML 135577 | 2002 | o | 74 |
| *Petrocephalus arnegardi* (paratype) (Odzala) | 5101 | [CUMV 88032](http://arctos.database.museum/guid/CUMV:Fish:88032) | CUML 135578 | 2002 | m | 73 |
| *Petrocephalus arnegardi* (paratype) (Odzala) | 5120 | [CUMV 88041](http://arctos.database.museum/guid/CUMV:Fish:88041) | CUML 135579 | 2002 | o | 85 |
| *Petrocephalus arnegardi* (non type) (Odzala) | 5122 | [CUMV 88043](http://arctos.database.museum/guid/CUMV:Fish:88043) | CUML 135580 | 2002 | m | 73 |
| *Petrocephalus arnegardi* (non type) (Odzala) | 5123 | [CUMV 88044](http://arctos.database.museum/guid/CUMV:Fish:88044) | CUML 135581 | 2002 | m | 66 |
| *Petrocephalus arnegardi (non type) (Odzala)* | 5124 | [CUMV 88045](http://arctos.database.museum/guid/CUMV:Fish:88045) | CUML 135582 | 2002 | m | 73 |
| *Petrocephalus arnegardi* (non type) (Odzala) | 5126 | [CUMV 88046](http://arctos.database.museum/guid/CUMV:Fish:88046) | CUML 135583 | 2002 | o | ? |
| *Petrocephalus arnegardi* (paratype) (Odzala) | 5158 | [CUMV 88052](http://arctos.database.museum/guid/CUMV:Fish:88052) | CUML 135584 | 2002 | m | 75 |
| *Petrocephalus arnegardi* (paratype) (Odzala) | 5159 | [CUMV 88053](http://arctos.database.museum/guid/CUMV:Fish:88053) | CUML 135585 | 2002 | m | 69 |
| *Petrocephalus arnegardi* (paratype) (Odzala) | 5197 | [CUMV 88063](http://arctos.database.museum/guid/CUMV:Fish:88063) | CUML 135586 | 2002 | o | 65 |
| *Petrocephalus arnegardi* (non type) (Odzala) | 5276 | [CUMV 88107](http://arctos.database.museum/guid/CUMV:Fish:88107) | CUML 135587 | 2002 | m | ? |
| *Petrocephalus arnegardi* (paratype) (Odzala) | 5377 | [CUMV 88123](http://arctos.database.museum/guid/CUMV:Fish:88123) | CUML 135588 | 2002 | m | 69 |
| *Petrocephalus arnegardi* (paratype) (Odzala) | 5395 | [CUMV 87830](http://arctos.database.museum/guid/CUMV:Fish:87830) | CUML 135589 | 2002 | o | 72 |
| *Petrocephalus arnegardi* (non type) (Odzala) | 5396 | [CUMV 88125](http://arctos.database.museum/guid/CUMV:Fish:88125) | CUML 135590 | 2002 | m | 74 |
| *Petrocephalus arnegardi* (paratype) (Odzala) | 5404 | [CUMV 87838](http://arctos.database.museum/guid/CUMV:Fish:87838) | CUML 135591 | 2002 | m | 90 |
| *Petrocephalus arnegardi* (non type) (Odzala) | 5423 | [CUMV 88128](http://arctos.database.museum/guid/CUMV:Fish:88128) | CUML 135592 | 2002 | m | 70 |
| *Petrocephalus arnegardi* (paratype) (Odzala) | 6133 | [CUMV 92390](http://arctos.database.museum/guid/CUMV:Fish:92390) | CUML 135594 | 2006 | m | 63 |
| *Petrocephalus arnegardi* (paratype) (Odzala) | 6134 | [CUMV 92390](http://arctos.database.museum/guid/CUMV:Fish:92390) | CUML 135595 | 2006 | m | 67 |
| *Petrocephalus arnegardi* (non type) (Yangambi) | JPS-10-511 | [BMNH 2013.8.29.125](http://www.nhm.ac.uk/research-curation/scientific-resources/collections/zoological-collections/zoology-specimen-database//?action=display&irn=2816740&RegRegistrationNumber=2013.8.29.125&RecordsPerPage=10&startAt=1) | CUML 135593 | 2010 | o | 49 |
| *Petrocephalus boboto* (holotype) (Yangambi) | JPS-10-426 | [CUMV 96774](http://arctos.database.museum/guid/CUMV:Fish:96774) | CUML 135596 | 2010 | o | 57 |
